# Notes from Past Meetings of the Lippe Society

**Published:** 1890-03

**Authors:** 


### NOTES FROM PAST MEETINGS OF THE LIPPE SOCIETY.

Dr. Lippe read a paper containing a translation § 154 of the *Organon*, with comments upon the ignoring of the principles of this paragraph by the mongrels, and a defense of the materia medica as it is against the efforts of the so-called "liberals" to revise it.

[If Dr. Lippe were living to-day he would find equally as much work for his pen against materia medica "revisers" as in the latter years of his life. If these men would only do as Dr. Lippe did, cure the sick, they would not be such blatant opponents of Homœopathy.]

Dr. Fellger remarked that were such efforts at revision successful there would be no Homœopathy at all.

[Dr. Fellger's life was spent in curing patients by adhering to Hahnemannian Homœopathy. He was able to appreciate its benefits, as he had been a surgeon in the German army and had become thoroughly disgusted with the vain efforts to cure disease which he saw while he occupied that position. Those who knew Dr. Fellger are alone able to do justice to his memory.]

Dr. Lippe, in his paper, mentioned the case of a man with typhoid fever who was constantly moaning and whining. It was not the deep, heavy moaning of Muriatic acid, but a whining constantly uttered. At the same time there was suppression of urine. Apis brought about a cure. Several years later the same man was taken with small-pox. The eruption failed to appear, and there was suppression of urine and constant whining. Apis was again given, and the next morning the pustules came out large as small grapes, urine flowed in large quantity, and there was no more whining.

Dr. Fellger, speaking of the effects of imagination, related the case of a blacksmith who sat down to dinner at which a custard was served, and which looked so like fecal matter that he immediately after vomited. It was impossible ever to mention the name of custard without his vomiting as a consequence. Another case was a woman who saw a criminal beheaded, and who soon after gave birth to a child with one hand amputated.

Dr. Lee knew of a case of a child being born circumcised, the mother having shortly before witnessed such an operation.

Dr. Fellger knew of a woman who, whilst pregnant, attempted to reach for a book on a shelf above her head. In so doing she brought down upon her head several other objects. When her next child was born it had a large swelling upon its head.

At a meeting at which Dr. Berridge was present a discussion arose in respect of the importance of avoiding perfumes about the sick-room. Dr. Berridge said that Dr. Wilson, of London, who was a most careful prescriber, had given Lachesis to a patient without perceptible effect. Being satisfied that it was the remedy, he searched for a cause for its non-action. He noticed that the patient had a handkerchief saturated with cologne. When this was taken away the next dose of Lachesis took effect.

Dr. Lippe asserted that violent emotions, like anger, will suspend the action of a remedy. He mentioned a case of toothache for which he gave Ratanhia$^{30}$ whilst the patient was smoking, and the pain was gone shortly after. We now, said Dr. Lippe, rarely see toothache.

I frequently have seen toothache precede tuberculosis. Now we scarcely ever see such a phenomenon ; but we see catarrh preceding the same troubles. If the catarrh be suppressed, then the tuberculosis will the sooner become manifest. This I have often seen in young girls.

Dr. Fellger, on the subject of Morphia, said that its use had largely increased in Germany. Some of those who took enough to develop its symptoms had certain involuntary motions, especially a backward ambling.

Dr. Berridge remembered that Picrotoxin will make an animal run backward. He had given that remedy to a dog which, while in a fit, ran backward, and cured him.

Dr. Fellger said he had once accidentally poisoned himself by scratching his finger with a pin charged with an arsenical preparation. He was troubled with dreams in which everything seemed dead—men, animals, plants, all seemed dead. Then came thirst.

Dr. Lippe said that Arsenicum metallicum was proved in 1850 by Dr. Duffield and himself, and published in the *Examiner*. Those who took it, and who had had syphilis many years before, had all the symptoms reappear. Their hair fell out, and they had chancres. These symptoms showed that Arsenicum metallicum was a remedy for some cases of syphilis.

Dr. Fellger had seen a case of syphilis in which the patient became so violent from the intense pain that he had to be held by four men. The symptoms all pointed to Arsenicum, which quieted the pain in a few hours, and by the next day it had entirely disappeared, and in about nine days the man was well. Dr. Lippe said that it was Dr. Duffield who observed that the Arsenicum pulse was more rapid in the morning than in the evening.

Diarrhœa.—Dr. C. Carleton Smith spoke of the epidemic of cholera which occurred in Chicago while he was practicing there. Patients would come into his office holding the hands upon the abdomen and fearing they would have an attack of diarrhœa immediately. They could not trust their bowels. Aloes would relieve all such cases, and they escaped the cholera afterward.

Dr. Lippe said the principal indication for Thrombidium in diarrhœa was much pain before and after stool and terrible tenesmus.

The stool is mostly mucus, with great straining. The attack comes on always after eating, no matter when. Drinking has nothing to do with it. Dr. Lippe had cured a very bad case of this kind of diarrhœa with Thrombidium, his attention being called to it by the indication, *diarrhœa occurring only after eating.*

Dr. Preston had had a case of chronic dysentery of only one or two stools a day. But after every stool was a discharge of jelly-like mucus. Kali-carb. cured.

Dr. Lippe added that Asarum has the same symptom.

Dr. Preston said that the symptom, jelly-like stool, could be found under Kali-carb. in Jahr's *Symptomen Codex.*

Dr. Fellger said that Aloes has the symptom, mucus passed after stool. The principal symptom of Aloes is jelly-like mucous stools.

Dr. Lippe had cured a case of diarrhœa in a horse following pneumonia by observing the symptom, mucous discharge from the wide-open anus. Phosphorus, one dose, was given, and the case was cured.

Dr. Clark related a case of diarrhœa with tenesmus and violent pain and a never-get-done feeling. Mercurius corrosivus relieved promptly when everything else failed.

Drs. Allen and Guernsey agreed that Mercurius solubilis, 6 CM potency, was a perfectly miraculous remedy for diarrhœa with tenesmus before, during, and after stool.

Constipation.—Dr. Long, of New Brunswick, said that in babies affected with constipation the doctor should see that the nurse does not use powders containing Lycopodium, as it causes constipation in babies.

Dr. Lippe said that when babies have constipation with painful stool, Veratrum would cure beautifully.

Dr. Long had cured a case of chronic constipation with Aloes; the indication being sense of weight in the pelvis before and after stool.

Dr. Lee had had a case of constipation in a child, for which he was unable to find any suitable remedy that would relieve until he noticed the *fan-like motion of the wings of the nose.* He gave Lycopodium and a cure promptly followed.

Disorders of Urination.—A case was reported of a man who had continual desire to urinate at night when lying upon the back. If he lay upon the side he was not troubled; but if he lay upon the back, he must get up at once. Pulsatilla was put down in Allen as the remedy having this symptom. It was given and cured him at once.

Dr. Mahlon Preston reported a case of a woman who had not passed any urine for three months.

Dr. Allen had had a similar case.

Dr. Fellger said that Zingiber is indicated where suppression of urine continues any length of time.

Dr. James spoke of the indication for Sarsaparilla: Can pass urine *only when standing up.* He had cured a little girl troubled for a long time with incontinence of urine at night during sleep. She had this symptom that she could not pass urine voluntarily except when standing up. Sarsaparilla was given, and cured immediately and permanently.

Dr. Guernsey said that Ferrum-phosphoricum has desire to pass urine whenever she moves about.

Dr. Guernsey had a case of a woman who for two years had a desire to urinate during the night. If she did not at once rise and pass water she had a violent headache. Gelseminum has the symptom, headache relieved by urination. Accordingly Gelseminum was given, which relieved very soon.

Nose-Bleed.—Dr. Fellger related the case of a man who died of nose-bleed notwithstanding the usual treatment of tampons used by his old-school physician.

Dr. Guernsey related that a cork factory took fire and burned down. Every one of the firemen was taken with nose-bleed. The doctor thought it might be accounted for as a proving of Carbo-veg. The doctor had cured cases of nose-bleed with Carbo-veg.

Dr. Constantine Lippe said that Dr. Swan claimed that Vipera acuatica Caranita was almost a specific for nose-bleed.

